# Inflammatory Response to Fine Particulate Air Pollution Exposure: Neutrophil versus Monocyte

**DOI:** 10.1371/journal.pone.0071414

**Published:** 2013-08-08

**Authors:** Xiaohua Xu, Silis Y. Jiang, Tse-Yao Wang, Yuntao Bai, Mianhua Zhong, Aixia Wang, Morton Lippmann, Lung-Chi Chen, Sanjay Rajagopalan, Qinghua Sun

**Affiliations:** 1 Davis Heart and Lung Research Institute, The Ohio State University, Columbus, Ohio, United States of America; 2 Division of Environmental Health Sciences, The Ohio State University, Columbus, Ohio, United States of America; 3 Department of Environmental Medicine, New York University School of Medicine, Tuxedo, New York, United States of America; 4 Division of Cardiovascular Medicine, College of Medicine, The Ohio State University, Columbus, Ohio, United States of America; University of Kentucky, United States of America

## Abstract

**Objectives:**

Studies have shown that chronic exposure to ambient fine particulate matter (less than 2.5 *µ*m in aerodynamic diameter, PM_2.5_) pollution induces insulin resistance through alterations in inflammatory pathways. It is critical to study how the immune system responds to this stimulant, which has been linked to cardiovascular and autoimmune diseases, but few studies have been focused on such involvement of both neutrophils and monocytes in a timely manner. We hypothesized that the neutrophil was involved in the inflammatory response to air pollution.

**Methods and Results:**

C57BL/6 mice were exposed to PM_2.5_ or filtered air (6 hours/day, 5 days/week) for 5, 14, and 21 days, respectively, in Columbus, OH. At the end of each of the exposure periods, we investigated the inflammatory response through flow cytometry, histology, intravital microscopy, and real-time PCR. PM_2.5_-exposed mice demonstrated a significant inflammatory response after 5 days of exposure. In the lung tissue and bronchoalveolar lavage fluid, monocytes/macrophages showed a transient response, while neutrophils showed a cumulative response. In addition, exposure to PM_2.5_ resulted in elevation of the monocyte chemoattractant protein 1 (MCP-1) cytokine, a monocyte/macrophage attractant in blood, at an early stage of exposure.

**Conclusions:**

These findings suggest that PM_2.5_ exposure induces the inflammatory responses from both macrophages and neutrophils involvement.

## Introduction

Over the past decades, research has furthered the understanding of the detrimental effects of air pollution on cardiovascular health [1], [2]. In particular, fine ambient particulate matter (less than 2.5 µm in aerodynamic diameter, PM_2.5_) is a collection of mostly inorganic pollutants that can penetrate deep into the lung [1]. Long-term exposure to PM_2.5_ has been associated with both increased risk for cardiovascular disease, diabetes-like symptoms, and cardiovascular health risks [3], [4]. All these impairments are associated with inflammation induced by air pollution. Nevertheless, few studies have been focused on the time-course of inflammatory responses induced by air pollution, and the underlying mechanisms that shapes the kinetics and extent of neutrophil and monocyte recruitment are still unclear.

Inflammation is a nonspecific immune response to any injury or attack, and is normally a self-limiting process [5]. Originally, the first key sign of inflammation is the recruitment of neutrophils from the vasculature into the injured tissue [6]. After neutrophils are recruited, they release cytokines and reactive oxygen species (ROS) to kill foreign bodies or infected cells [7], and these cytokines were thought to be responsible for monocyte recruitment [6]. Therefore, neutrophils are thought to function as the first responders, while monocytes, which could differentiate into macrophages, to be secondary responders. However, a number of studies have shown that macrophage recruitment may be independent of neutrophil cytokine release, while macrophages may even contribute to the recruitment of neutrophils [6], [8].

Macrophages are able to exhibit both pro- and anti-inflammatory characteristics. Classically activated macrophages are stimulated by a variety of cytokines, such as CCR1, CCR2 and CCR4, physiological conditions, such as hypoxia and abnormal matrices, and foreign stimuli, such as parasites and bacteria [9], [10]. Upon activation, these macrophages produce high concentrations of inflammatory cytokines, such as IL-12, and reactive oxygen species (ROS) [11]. Pro-inflammatory macrophages also dictate the actions of other cells. Macrophage release of the Fas-ligand causes apoptosis of neutrophil cells, while TNF-α causes the death of tissue cells [12], [13]. In addition to the pro-inflammatory response, macrophages can also be stimulated into differentiating into anti-inflammatory cells (M2). Anti-inflammatory cell migration occurs when tissues release Th2 cytokines such as IL-4 and IL-13 [14]. Anti-inflammatory cells function to alleviate inflammation in a particular area by reducing the production of Nos2 and IL-12, while increasing the expression of IL-10 [14]. Functionally, anti-inflammatory macrophages release fibronectin and collagen I when co-cultured with myofibroblasts [15]. Anti-inflammatory macrophages further protect the matrix by producing transglutaminase, which protects bodily polymers from being broken down [16]. In short, anti-inflammatory macrophages serve to reform and protect the cellular matrix to resolve the inflammatory response.

Recently, it has been discovered that neutrophils can exhibit properties similar to polarizations of macrophages. Neutrophils seem capable of differentiating into two states that are capable of either aiding or hindering tumor formation [17]. Activation of anti-inflammatory neutrophils resulted in the increase production of neutrophil chemoattractants. Conversely, neutrophils will take up a pro-inflammatory role when they are exposed to TGF-β [17]. Therefore, we hypothesized that exposure to PM_2.5_ leads an inflammatory response from both macrophages and neutrophils.

## Materials and Methods

### Animals

Three-week-old male C57BL/6 mice were purchased from Jackson Laboratories (Bar Harbor, MA). All mice were given 7 days to adjust to their new environment before PM_2.5_ exposure. All mice were fed rodent standard laboratory chow and allowed to eat *ad libitum* throughout the duration of the study. The mice were housed on a 12-hour light-dark cycle in a temperature-controlled room at 22°C. NIH guidelines for the care and use of laboratory animals were strictly followed, and all experiments were approved by the Institutional Animal Care and Use Committee at The Ohio State University.

### PM_2.5_ Exposure

Mice were randomly assigned to 6 experimental groups and were exposed to either ambient concentrated PM_2.5_ or filtered air (FA) for 6 hours/day, 5 days/week for 5, 14, and 21 days, respectively, from Dec. 14, 2009 to Jan. 4, 2010, in Columbus, Ohio, in a trailer-mounted exposure system, “Ohio Air Pollution Exposure System for Interrogation of Systemic Effects (OASIS) 1″ [18], [19], [20].

### Intravital Microscopy

At the end of the exposure, the mice were anesthetized intraperitoneally by a mixture of ketamine (100 mg/kg) and xylazine (20 mg/kg). The mesenteric tissue was exteriorized. The tissue was superfused with preheated Ringer’s lactate on an optical mount. The number of rolling and adherent cells was determined with a 40×/0.80-W water-immersed objective using a Nikon Eclipse FN1 microscope (Nikon, Tokyo, Japan) in a 100-µm vessel length per 30 seconds per image field (1.57×10^5^ µm^2^), and the data presented were averaged from 10 vessels per mouse. Cells that remained stationary for at least 5 seconds were considered “adherent” cells [4], [18].

### Measurement of Blood Inflammatory Biomarkers

After the mice have been fasted overnight, blood was collected and the serum was isolated and stored at −20°C until further use. Cytokine levels were determined by Cytometric Bead Array (BD Biosciences). Sera were incubated with beads specific for tumor necrosis factor (TNF), interferon γ (IFN-γ), monocyte chemoattractant protein 1 (MCP-1), interleukin 6 (IL-6), IL-10, and IL-12p70 according to the manufacturer’s instructions.

### Flow Cytometry

At the end of the exposure, bronchoalveolar lavage fluid (BALF) was harvested. Cells were stained with anti-Ly6G coupled with phycoerythrin (PE) and anti-F4/80 coupled with allophycocyanin (APC) antibodies from BioLegend (San Diego, CA) to detect neutrophil and macrophage populations, respectively. The cells were analyzed on a LSR II flow cytometer (BD Biosciences). As negative controls, cell aliquots were incubated with isotype-matched rat IgGs under the same conditions.

### Immunohistochemistry

Lung and epididymal fat were harvested and fixed in 10% neutral buffer formalin. Immunohistochemical staining was performed as previously described [18], [21]. The slices were stained with rat anti-mouse NIMP-R14 (Abcam, Cambridge, MA) to identify neutrophil populations, and rat anti-mouse F4/80 (AbD Serotec, Raleigh, NC) to identify macrophage population. Images of the slides were captured at 200× magnification with a Nikon Eclipse FN2 microscope equipped with Metamorph software. All measurements were conducted in a double-blinded manner by two independent investigators.

### Real-time PCR

Epididymal fat was harvested and homogenized by TRIzol Reagent (Invitrogen, Carlsbad, CA). Total RNA was then converted into cDNA using the High Capacity cDNA Reverse Transcription Kit (Applied Biosystems, Foster City, CA). The quantification of gene expression was determined by real-time PCR. All reactions were performed under the following conditions: 50°C for 2 minutes, 95°C for 10 minutes, 40 cycles of 95°C for 15 seconds, and 60°C for 1 minute. The primers for mouse *IL-6*, *Nos2*, *Tnf-α*, *Arg-1*, *IL-10*, and *β–actin* are showed in Table 1. Beta–actin was used as the control gene and all data are represented as relative mRNA expression on gene expression.

**Table 1:** Primers Used for Real-time PCR.

Primer	Forward oligonucleotides	Reverse oligonucleotides
*IL-6*	5′-TGGCTAAGGACCAAGACCATCCAA-3′	5′-AACGCACTAGGTTTGCCGAGTAGA-3′
*Nos2*	5′-TCTTTGACGCTCGGAACTGTAGCA-3′	5′-ACCTGATGTTGCCATTGTTGGTGG-3′
*TNF-α*	5′-TCTCATGCACCACCATCAAGGACT-3′	5′-TGACCACTCTCCCTTTGCAGAACT-3′
*Arg-1*	5′-TGGCTTTAACCTTGGCTTGCTTCG-3′	5′-CATGTGGCGCATTCACAGTCACTT-3′
*IL-10*	5′-TTGCTCTTGCACTACCAAAGCCAC-3′	5′-AGTAAGAGCAGGCAGCATAGCAGT-3′
*β-actin*	5′-TGTGATGGTGGGAATGGGTCAGAA-3′	5′-TGTGGTGCCAGATCTTCTCCATGT-3′

### Human Neutrophil Isolation

Polymorphonuclear cells were isolated from human peripheral blood by Lympholyte®-poly kit (Cedarlane). Human peripheral blood was provided by American Red Cross Blood Services Central Ohio Region (ARCBSCOR). This study was approved by The Ohio State University Institutional Review Board (IRB). Human neutrophils were purified by negative selection using EasySep™ Magnet according to the manufacturer’s instructions (Stemcell Technologies).

### Cell Migration Assay

Cell migration was assayed using a modified Boyden chamber consisting of a 48-well microchamber (Neuro Probe, Gaithersburg, MD). Briefly, 10 mg of adipose tissue was incubated in 500 µl of serum-free RPMI 1640 for 16 hours. After centrifugation, 26 µl of the conditioned media was added to the lower wells, and a 5-µm pore-size polyvinylpyrrolidone-free polycarbonate membrane (Neuro Probe, Gaithersburg, MD) was placed between the lower wells and upper wells. RAW264.7 cells (ATCC #TIB-71) were suspended in culture medium at 1×10^7^ cells/ml, and 50 µl aliquot of the cell suspension was then placed into the upper wells. The chamber was incubated for 4 hours at 37°C in a humidified incubator with 5% CO_2_. After incubation, the cells adherent to the upper surface of the membrane were removed by scraping with a rubber blade. The cells that had migrated through the membrane and adherent to the underside of the membrane were fixed and stained with Hema-3 system (Fisher Scientific, Pittsburgh, PA), mounted, and the cell migration was quantified by counting under light microscope. The results are presented as migrated cell number/five high power fields (HPF) that were chosen randomly. The cell migration assay of human neutrophils followed the same procedure as RAW264.7 cells, except that the neutrophils were suspended in culture medium at 1×10^6^ cells/ml. After incubation, the cells migrated through the membrane were counted in the lower wells of the Boyden chamber by Countess® Automated Cell Counter (Life Technologies).

### Statistical Analysis

Data are expressed as mean ± s.e. unless otherwise indicated. The experimental results were examined by analysis of variance. All the analyses were performed using Graphpad Prism v5.0 (GraphPad Software, San Diego, CA). In all cases, a *P* value of <0.05 was considered statistically significant.

## Results

### Exposure Characterization

The mean daily PM_2.5_ concentration at the study site in Columbus, OH, was 12.1 (s.d., 5.9) *µ*g/m^3^. The mean concentration of PM_2.5_ in the exposure chamber during the 30 hours/week of exposure was 143.8 *µ*g/m^3^ (∼12-fold concentration from ambient PM_2.5_ level).

### Effect of Exposure to PM_2.5_ on Adherent Leukocyte and Rolling in Inflamed Venules

**Figure 1:**
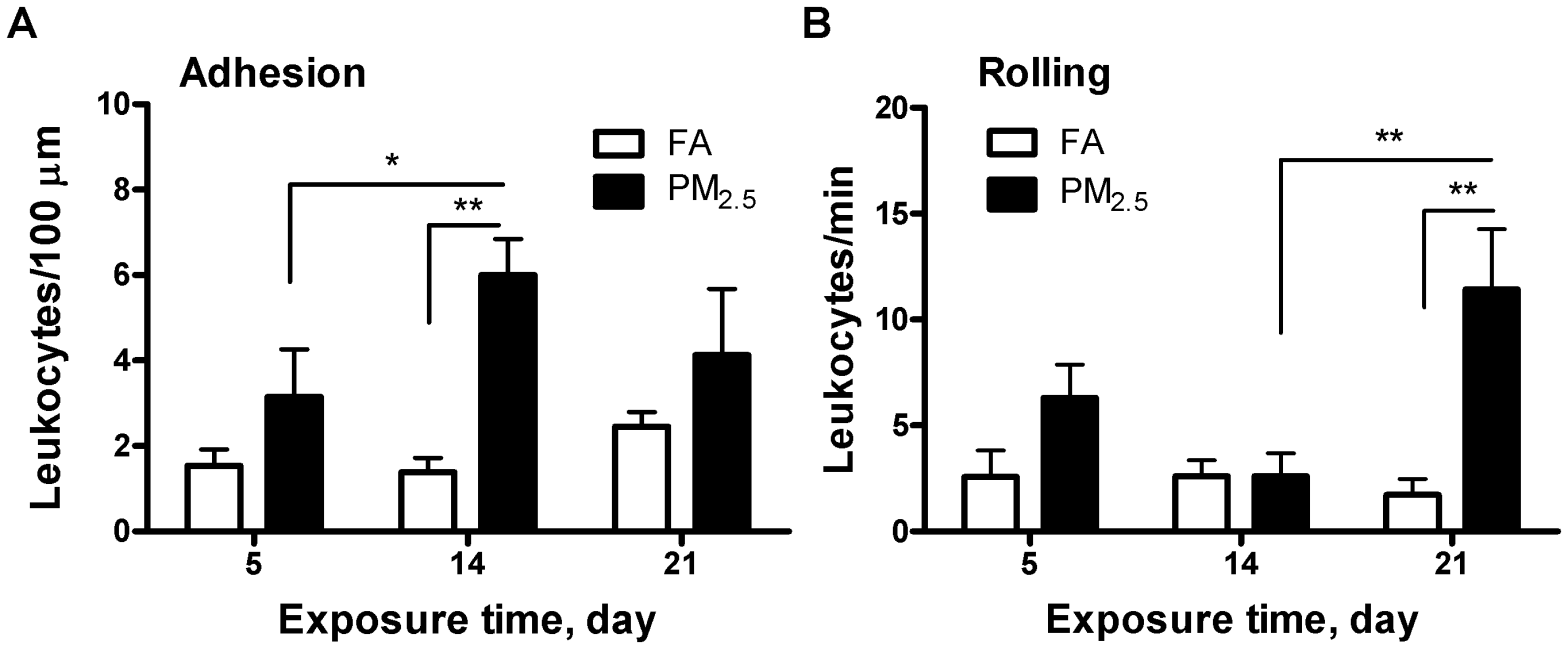
Effect of PM_2.5_ exposure on systemic inflammation in microvasculature measured by intravital microscopy. Leukocyte adhesion (A) and rolling flux (B) in mesenteric circulation. N = 6. *P<0.05, **P<0.001.

At the end of the exposure to PM_2.5_ or FA, intravital microscopy was performed to evaluate the number of rolling and adherent cells, as an index of recruitment into tissue depots. As demonstrated in Figure 1A–B, the results showed that PM_2.5_ exposure resulted in significant increases in both adherent cells after 14-day PM_2.5_ exposure and rolling cells after 21-day PM_2.5_ exposure. Adherent cells were elevated by a four-fold increase (6.00 in PM_2.5_ *vs.* 1.39 in FA group) after 14 days, while rolling cells were elevated by a six-fold increase (11.43 in PM_2.5_ *vs.* 1.73 in FA group) after 21 days. The number of adherent cells in the 14-day PM_2.5_-exposed mice was also significant higher than that in the 5-day PM_2.5_-exposed mice, whereas more rolling cells in 21-day PM_2.5_-exposed mice were observed than in 14-day PM_2.5_-exposed mice.

### Effect of Different Times of PM_2.5_ Exposure on Cytokine Production

**Figure 2:**
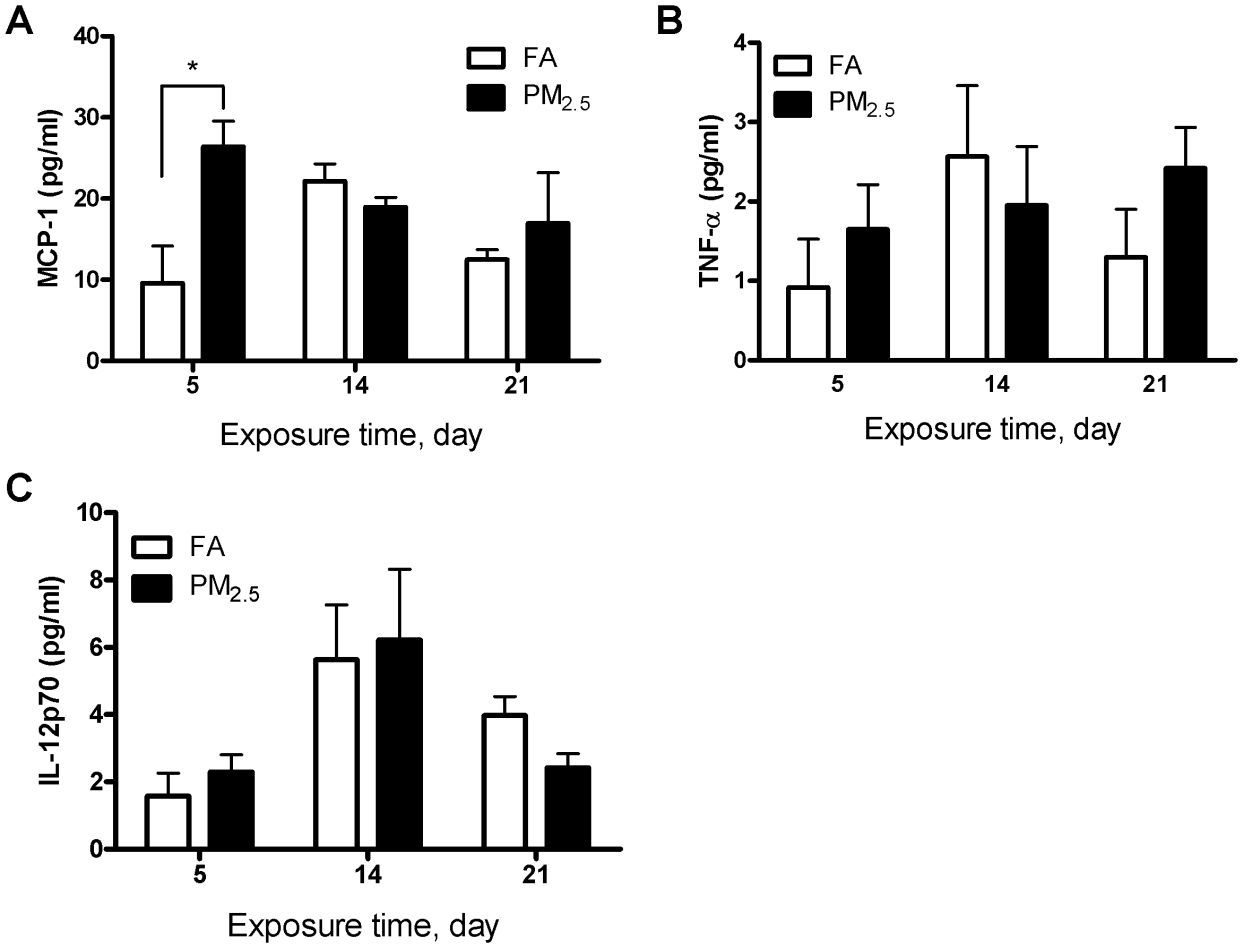
Effect of PM_2.5_ exposure on inflammatory cytokines in circulation. (A) MCP-1, (B) TNF-α, (C) IL-12p70. N = 6. *P<0.05.

After the end of the exposures, peripheral blood was collected, spun, and serum was stored at −80°C for the analysis of cytokines. As shown in Figure 2, MCP-1 was found to be significantly elevated after 5 days of exposure (26.37 pg/ml in PM_2.5_ *vs.* 9.58 pg/ml in FA). Moreover, this elevated protein expression was not sustained past the 5 initial days of exposure, and was reduced after 21 days. There were no significant changes between the FA- and PM_2.5_-exposed groups for IL-12 or TNF-α concentrations in the blood, while the levels of IFN-γ, IL-6 and IL-10 were too low to be detectable.

### Effect of Different Times of PM_2.5_ Exposure on Gene Expression

**Figure 3:**
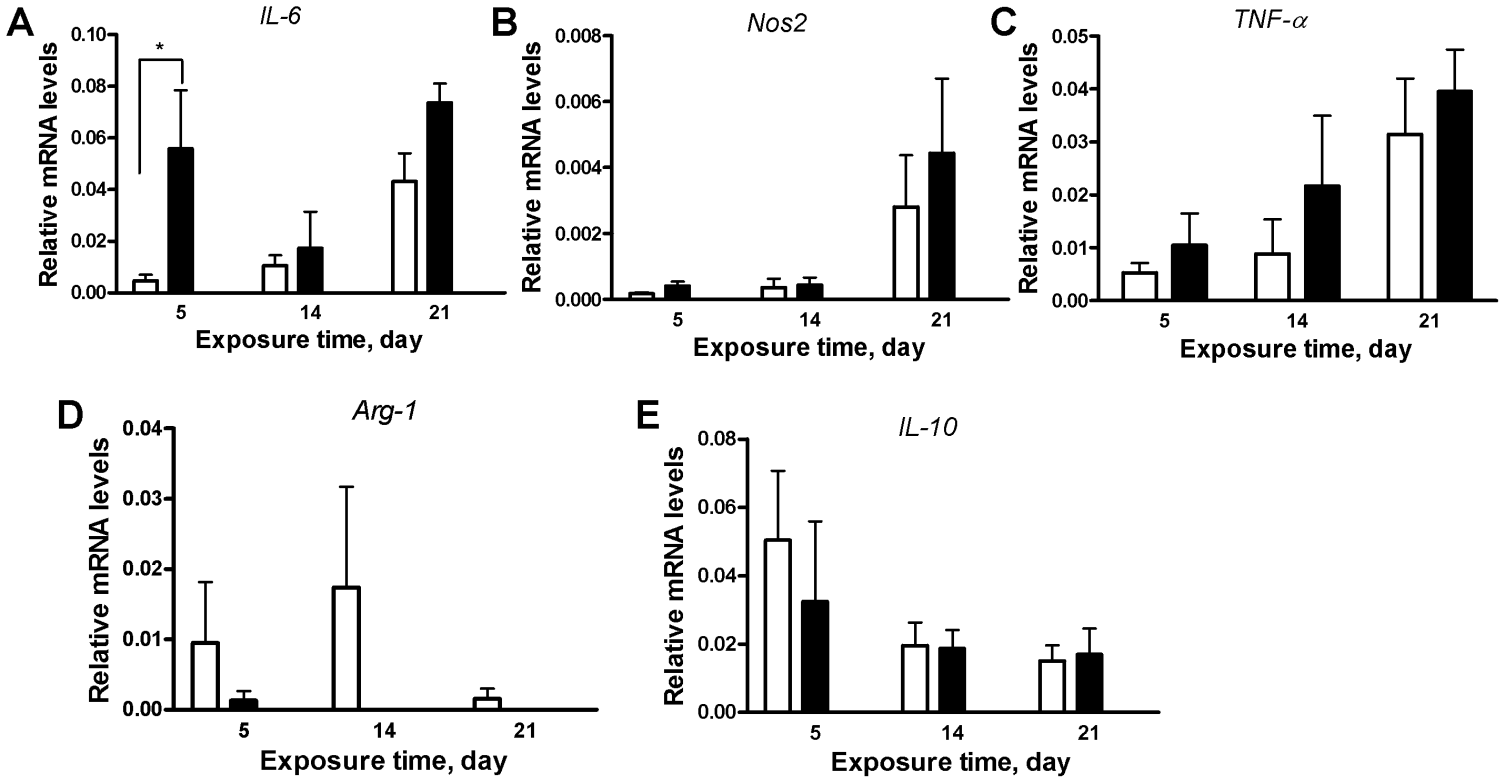
Effect of PM_2.5_ exposure on gene expression for macrophage phenotypic changes in visceral adipose tissue. (A), *IL-6*, (B) *Nos2*, (C) *TNF-α*, (D) *Arg-1*, (E) *IL-10*. Open bars represent FA group, and solid bars represent PM_2.5_ group. N = 6. *P<0.05.

As shown in Figure 3, there were no significant differences in the mRNA levels of *Nos2*, *TNF-α*, *Arg-1*, or *IL-10* in the epididymal adipose depots. Nevertheless, there was a 12-fold increase in the expression of *IL-6* during the first five days of PM_2.5_ exposure when compared with the corresponding FA group. These findings suggest that acute inflammation is present after just 5 days of exposure. Furthermore, macrophage cells can be rapidly recruited to an inflamed site.

### Effect of Different Times of PM_2.5_ Exposure on Inflammatory Cell Infiltration

**Figure 4:**
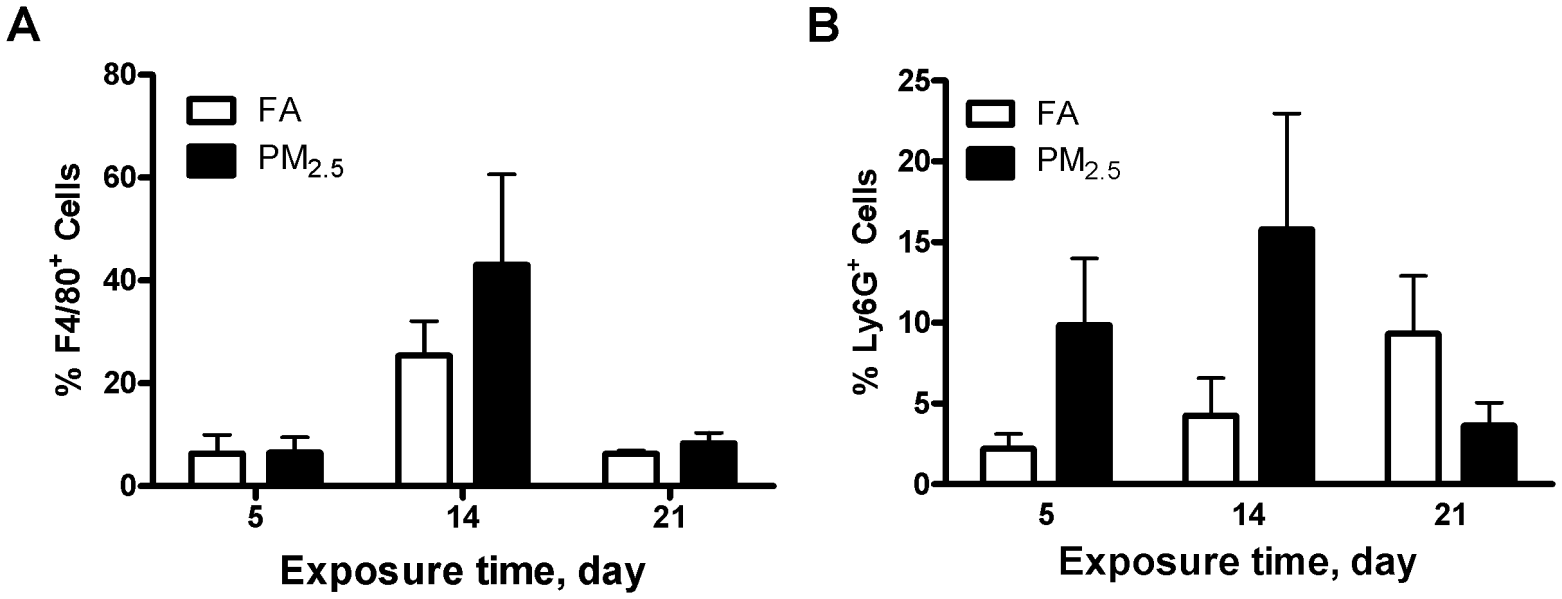
Effect of PM_2.5_ exposure on macrophages and neutrophils in bronchoalveolar lavage fluid (BALF). Flow cytometric analysis of F4/80 (A) or Ly6G (B) in the BALF. N = 6.

**Figure 5:**
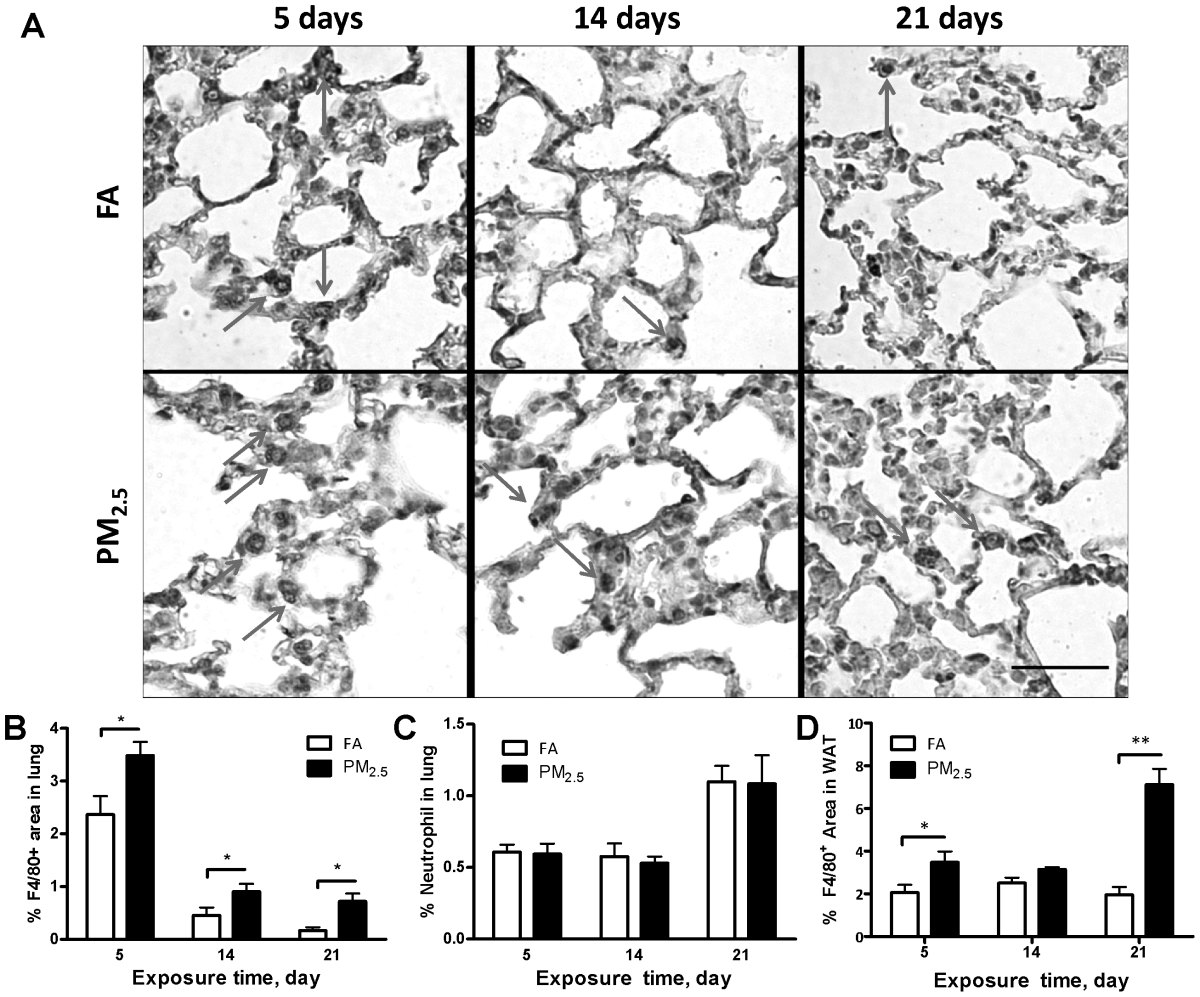
Effect of PM_2.5_ exposure on macrophages and neutrophils in the lung and visceral adipose tissue. Representative images (A) and statistical analysis (B) of the immunohistochemical staining for F4/80^+^ of macrophages in the lung. Statistical analysis of the immunohistochemical staining for neutrophils (NIMP-R14^+^) in the lung (C) and F4/80^+^ of macrophages in the epididymal adipose tissue (D). Arrows point to positive staining. Scale bar, 100 µm. N = 6. WAT, white adipose tissue. *P<0.05, **P<0.001.

To specifically investigate the infiltration of macrophages or neutrophils, we measured the macrophages and neutrophils in the BALF by flow cytometry. As shown in Figure 4A–B, there were no significant differences in either macrophage or neutrophil recruitment in BALF. Recruitment was also analyzed through immunohistochemistry with macrophage (F4/80) and neutrophil (NIMP-R14) markers. In lung tissue, macrophage recruitment was observable within the first 5 days of exposure, indicating that macrophages can be quickly recruited to an area of injury during acute inflammation (Figure 5A–B). Surprisingly, neutrophils did not exhibit such clear recruitment or activation properties (Figure 5C). We also investigated the inflammatory responses in the adipose tissue. As shown in Figure 5D, macrophage recruitment in the epididymal adipose tissue was generally elevated during the study, peaking after 21 days of exposure. Neutrophil recruitment was not analyzed for the epididymal fat because previous studies indicate little, if any, responses of neutrophils in that area. This findings suggest that there is widespread inflammatory response by both neutrophil and macrophage cells; however, how do they interact still needs further investigation.

### Chemotaxis

**Figure 6:**
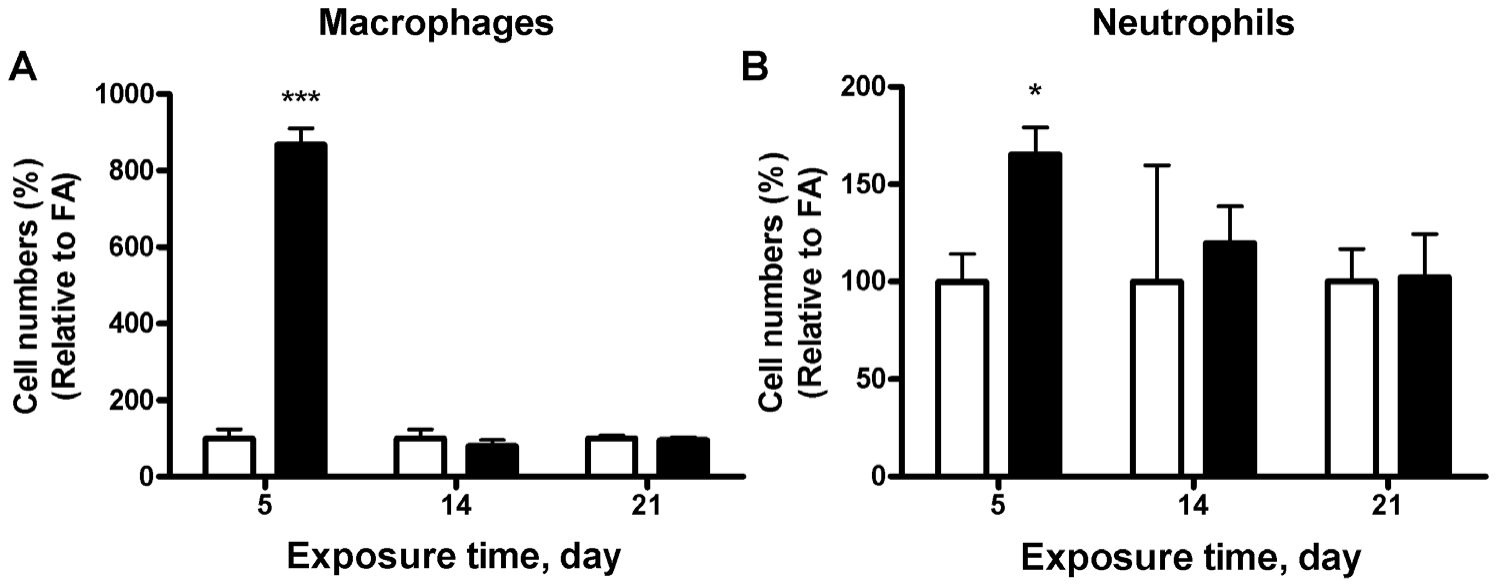
Effect of PM_2.5_ exposure on chemotactic migration. Chemotactic migration of macrophages (A) and neutrophils (B). N = 6. ***P<0.0001 vs. FA group.

Adipose tissue macrophages are thought to represent key cellular mediators of adipose tissue inflammatory response and insulin resistance development. To investigate potential alteration in chemokine factors within visceral adipose tissue, we evaluated the chemotactic ability of conditioned media derived by culturing visceral adipose tissue. The migratory capacity of monocytes or neutrophils was tested by quantifying the numbers of monocytes or neutrophils moving towards the chamber containing conditioned media. The numbers of monocytes and neutrophils migrated towards conditioned media from visceral fat were significantly increased in the conditioned media from the mice exposed to PM_2.5_ for 5 days, when compared with the corresponding FA-exposed group (Figure 6A–B). Nonetheless, the numbers of monocytes and neutrophils sustained were low in the conditioned media from the mice exposed to PM_2.5_ for 14 and 21 days as in the corresponding FA-exposed group. These findings suggest that the cytokines that were released from the adipose tissue could be shortly induced by PM_2.5_ exposure for 5 days, and thereafter remain at low levels.

## Discussion

In this study, we evaluated the effect of different time-courses of exposure to PM_2.5_ air pollution on systemic inflammatory response, monocyte and neutrophil infiltration and aggregation in tissues in mice. To our knowledge, this is the first to study the time-course response to PM_2.5_ air pollution that indicated that monocytes/macrophages and neutrophils are involved in this process and changes in different compartments with PM_2.5_ exposure. In this study we evaluated the systemic inflammatory response towards PM_2.5_ over a period of three weeks. PM_2.5_ exposure induces inflammatory responses in the lung and visceral adipose tissue.

A number of studies have revealed associations of exposure to PM_2.5_ with systemic pro-inflammatory responses in humans and animals [18], [22], [23], [24]. In addition, accumulating studies have highlighted the innate immune mechanisms play critical roles that in the pathophysiological abnormalities [25], [26]. Recently, the temporal order of immune responses in inflammation has been called into question. Studies have demonstrated that the original model of bimodal immune response may not be necessarily accurate [6]. In this study, systemic chronic inflammation as indicated by leukocytes recruitment in the microcirculation was observable and significant within 3 weeks of exposure, with markers for macrophage migration being detectable shortly after 5 days of exposure. Data from our study on neutrophil and macrophage populations support the growing body of evidence that possibly challenge the bimodal immune response. Additionally, chronic inflammation resolution will also rely on understanding immune cell migration. Understanding the mechanisms behind macrophage and neutrophil recruitment is crucial in any attempt to control chronic inflammation.

Acute inflammation is a natural way for the immune system to clear away pathological agents. Although the immediate inflammatory response plays an important role in host defense, uncontrolled inflammation can contribute to a disease progression, such as pulmonary disease [27]. Therefore, it is of interest to identify environmental factors that may modify innate immune responsiveness. According to the bimodal immune response model, neutrophil cells should be recruited to an area of injury first, where they activate and then releases cytokines to attract other immune cells, such as macrophage cells [6]. Therefore, populations of neutrophil cells should be quickly elevated, followed by populations of macrophage cells becoming elevated. In contrast, our data support the growing evidence that macrophages can be recruited without neutrophil cell stimulation. The brief elevation of IL-6, a cytokine upregulated in obesity and closely associated with systemic endothelial dysfunction [28], in the adipose tissue after five days suggests the significant acute inflammation after that exposure [29]. Up to 35% of plasma IL-6 originates from adipose tissue, and other cytokines such as IL-10 and TNF-α are also reported to be elevated in obesity and linked to a wide range of metabolic complications [30], [31]. These findings are important because inflammatory mechanisms play a pivotal role not only in early stages of endothelial dysfunction, but also destabilization of advanced atherosclerotic plaques that precipitate acute cardiovascular events that may be induced by ambient particulate air pollution exposure [4], [32], [33]. Surprisingly, neutrophil response was not significantly changed during the short-term exposure in any of the tissues tested. However, during that same time period, macrophage populations were significantly elevated in both the lung and visceral adipose tissue. These data suggest two possible interpretations. Either the data are similar to those found by Henderston *et al* [6], when monocyte populations were found in tissue even in neutrophil depleted mice, or that the inflammation has progressed to the resolution phase, whereby the present macrophage cells are clearing away the apoptotic neutrophil cells.

This data mirror other data that showed acute inflammatory responses after approximately one week of exposure. In the study by Martin *et al.* [34], lung histology was performed on mice after 7 days after exposure to air pollution particles. We also found visible signs of inflammation within the lungs and alveolar tract.

Chronic inflammation is a significant biological problem, and previous studies have demonstrated that exposure to air pollution classified as PM_2.5_ is consistently capable of producing such a problem [4], [18]. The data from intravital microscopy indicates a continual elevation in leukocyte recruitment in the microcirculation. This continual elevation suggests that these mice were experiencing chronic inflammation within just a few weeks. Continual elevation of macrophage recruitment in the epididymal fat also supports this conclusion. Furthermore, initial inflammation in the lungs and other tissues was followed by primarily inflammation in distant tissue, supporting the idea that PM_2.5_ is not only capable of affecting the lungs, but can be responsible for the circulation of its constituents to other parts of the body.

In summary, our data suggest that PM_2.5_ exposure may induce pro-inflammatory responses from macrophages and neutrophils. These findings suggest that that even low levels of ambient air PM_2.5_ can have an important public health impact on human populations.
